# Impact of visceral fat on the prognosis of coronavirus disease 2019: an observational cohort study

**DOI:** 10.1186/s12879-021-06958-z

**Published:** 2021-12-10

**Authors:** Hiroaki Ogata, Masahiro Mori, Yujiro Jingushi, Hiroshi Matsuzaki, Katsuyuki Katahira, Akiko Ishimatsu, Aimi Enokizu-Ogawa, Kazuhito Taguchi, Atsushi Moriwaki, Makoto Yoshida

**Affiliations:** 1 Department of Respiratory Medicine, National Hospital Organization Fukuoka National Hospital, 4-39-1 Yakatabaru, Minami-ku, Fukuoka, 811-1394 Japan; 2 Department of Radiology, National Hospital Organization Fukuoka National Hospital, 4-39-1 Yakatabaru, Minami-ku, Fukuoka, 811-1394 Japan; 3 Department of Pediatrics, National Hospital Organization Fukuoka National Hospital, 4-39-1 Yakatabaru, Minami-ku, Fukuoka, 811-1394 Japan

**Keywords:** Severe acute respiratory syndrome coronavirus 2, Coronavirus disease 2019, Computed tomography, Obesity, Visceral adipose tissue

## Abstract

**Background:**

Clarification of the risk factors for coronavirus disease 2019 (COVID-19) severity is strongly warranted for global health. Recent studies have indicated that elevated body mass index (BMI) is associated with unfavorable progression of COVID-19. This is assumed to be due to excessive deposition of visceral adipose tissue (VAT); however, the evidence investigating the association between intra-abdominal fat and COVID-19 prognosis is sparse. We therefore investigated whether measuring the amount of intra-abdominal fat is useful to predict the prognosis of COVID-19.

**Methods:**

The present study enrolled 53 consecutive cases of COVID-19 patients aged ≥ 20 years with chest computed tomography (CT) scans. The VAT area, total adipose tissue (TAT) area, and VAT/TAT ratio were estimated using axial CT images at the level of the upper pole of the right kidney. Severe COVID-19 was defined as death or acute respiratory failure demanding oxygen at ≥ 6 L per minute, a high-flow nasal cannula, or mechanical ventilation. The association of VAT/TAT with the incidence of progression to a severe state was estimated as a hazard ratio (HR) using Cox regression analysis. To compare the prediction ability for COVID-19 disease progression between BMI and VAT/TAT, the area under the receiver operating characteristic curve (AUC) of each was assessed.

**Results:**

A total of 15 cases (28.3% of the whole study subjects) progressed to severe stages. The incidence of developing severe COVID-19 increased significantly with VAT/TAT (HR per 1% increase = 1.040 (95% CI 1.008–1.074), *P* = 0.01). After adjustment for potential confounders, the positive association of VAT/TAT with COVID-19 aggravation remained significant (multivariable-adjusted HR = 1.055 (95% CI 1.000–1.112) per 1% increase, *P* = 0.049). The predictive ability of VAT/TAT for COVID-19 becoming severe was significantly better than that of BMI (AUC of 0.73 for VAT/TAT and 0.50 for BMI; *P* = 0.0495 for the difference).

**Conclusions:**

A higher ratio of VAT/TAT was an independent risk factor for disease progression among COVID-19 patients. VAT/TAT was superior to BMI in predicting COVID-19 morbidity. COVID-19 patients with high VAT/TAT levels should be carefully observed as high-risk individuals for morbidity and mortality.

**Supplementary Information:**

The online version contains supplementary material available at 10.1186/s12879-021-06958-z.

## Introduction

Severe acute respiratory syndrome coronavirus 2 (SARS-CoV-2) is still causing a global pandemic of coronavirus disease 2019 (COVID-19). COVID-19 is characterized by high rates of development of acute respiratory distress syndrome, requirement for intensive care, and mortality [1]; it is thus a considerable health burden worldwide. Understanding the factors contributing to disease severity and complications of COVID-19 is of key importance to identify pathways for targeted prevention and a public health priority [2].

Overweight and obesity, defined as abnormal or excessive fat accumulation, are well-established risk factors for the development of noncommunicable diseases [3]. Of note, it has been demonstrated that visceral fat, but not so much subcutaneous fat accumulation, promotes systemic inflammation and impairs health [4]. Recent reports have suggested that an increase in body mass index (BMI) was associated with COVID-19 disease severity and mortality [5, 6]. However, the evidence of the influence of excessive visceral fat deposition on COVID-19 is sparse.

Based upon these considerations, the present study was conducted as a longitudinal cohort survey to evaluate the associations between intra-abdominal fat and disease progression among subjects with COVID-19.

## Methods

### Study population

**Fig. 1:**
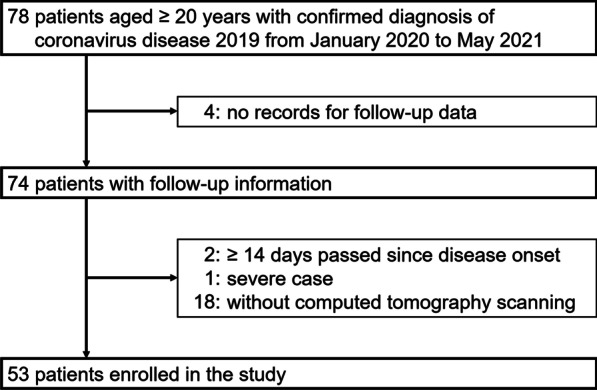
Selection of subjects

This study was conducted through a retrospective review of medical records at National Hospital Organization Fukuoka National Hospital, which serves residents of the southern area of Fukuoka City, situated on the northern shore of Kyushu Island in Japan. Since severe or fatal COVID-19 is extremely rare in adolescents and youths [7–9], we reviewed 78 patients aged ≥ 20 years with a diagnosis of COVID-19 confirmed by positive reverse-transcription polymerase chain reaction tests or rapid antigen tests for SARS-CoV-2 between January 1, 2020, and May 31, 2021. Among them, we excluded 4 patients with no available medical records for follow-up data, 2 patients admitted later than 14 days after disease onset, 1 patient who was already in a severe state on admission, and 18 patients who did not undergo computed tomography (CT) scans; the remaining 53 cases were enrolled in the present study (Fig. 1). All subjects were naïve to vaccination against SARS-CoV-2.

### Assessment of the abdominal fat component

CT examinations were performed with a 160-slice multidetector CT scanner (Aquilion Lightning, Canon Medical Systems, Otawara, Japan). For each patient, visceral adipose tissue (VAT) and subcutaneous adipose tissue (SAT) areas were semiautomatically segmented on an axial CT image at the level of the upper pole of the right kidney, which was lower than the costophrenic angle level, using dedicated software (AZE Virtual Place, Canon Medical Systems, Otawara, Japan). Using the same image and software, waist circumference was also automatically estimated at the level of the upper pole of the right kidney. The total adipose tissue (TAT) area was calculated as the sum of the VAT and SAT areas. The proportion of the VAT area was defined as VAT/TAT. A radiologic technologist without prior knowledge of the clinical data performed the procedure. When dividing the study subjects into three groups based on the tertile distribution of VAT/TAT, the cutoff values were as follows: lowest, ≤ 48.9% (n = 17); middle, 49.0–66.1% (n = 18); and highest, ≥ 66.2% (n = 18). In the study population, there were 8 cases whose axial CT data were consecutive to the navel or pelvic level; the aforementioned radiologic variables, namely, VAT, SAT, TAT, and waist circumference at the navel level were also obtained to assess the correlations with those at the upper pole of the right kidney.

### Clinical evaluations

For each case, respiratory physicians reviewed the medical records and assessed the demographic and clinical characteristics: age, sex, height, weight, smoking status, medical history, and clinical course. Severe COVID-19 was defined as death or acute respiratory failure demanding oxygen at ≥ 6 L per minute, a high-flow nasal cannula, or mechanical ventilation to maintain oxygen saturation (SpO_2_) ≥ 90%. Critical cases were limited to those meeting any of the following criteria: (i) death or (ii) acute respiratory failure requiring oxygen at ≥ 10 L per minute or mechanical ventilation to maintain SpO_2_ ≥ 90%. Height and weight were self-reported. After excluding 1 case with no available data on height or weight, BMI (kg/m^2^) was calculated as weight divided by squared height for 52 cases. According to the World Health Organization recommendation on treatment for obesity in the Asia–Pacific region [10], obesity and underweight were defined as BMI ≥ 25.0 kg/m^2^ and BMI < 18.5 kg/m^2^, respectively. Smoking status was dichotomized as smokers and never smokers. Hypertension and diabetes were defined as current treatment with antihypertensive agents or antidiabetic medication (oral hypoglycemic agents, injectable glucagon-like peptide analogs, or insulin), respectively.

### Statistical analysis

SAS University Edition software version 9.4 (SAS Institute, Cary, NC, USA) was used to perform all statistical analyses. A two-sided *P* < 0.05 was considered to indicate statistical significance. Demographic and clinical characteristics were shown as the mean values with standard deviance for continuous variables or frequencies for dichotomous ones. To test the reliability of the radiological variables (VAT, SAT, TAT, and waist circumference) obtained at the upper pole of the right kidney, Pearson’s correlation coefficients with those at the navel were calculated. The associations of obesity-related factors (BMI, VAT, SAT, TAT, VAT/TAT, and waist circumference) with the incidence of developing severe or critical COVID-19 were estimated as crude and multivariable-adjusted hazard ratios (HRs) with 95% confidence intervals (95% CIs) in Cox proportional hazard models. In a multivariate analysis, adjustment was made for age, sex, smoking status, hypertension, and diabetes. To assess the relationship between VAT/TAT levels and the risk of developing severe or critical COVID-19, we divided the subjects into three groups using the tertile distribution of VAT/TAT, as described above. The cumulative incidence in each group was estimated using the Kaplan–Meier method. The linear trend and the difference across the groups were evaluated using the relevant Cox models. In the same way, the trends in the BMI risk for the incidence of severe or critical COVID-19 were analyzed by classifying the subjects as underweight, normal, or obese. To compare the prediction ability for disease progression of COVID-19 between the models adjusted for BMI and VAT/TAT, the receiver operating characteristic (ROC) curve for each model was generated. The area under the ROC curve (AUC) was compared between two models using DeLong’s method [11]. The robustness of the main results was verified by sensitivity analyses of each subgroup with or without a smoking history.

### Ethical considerations

The study was approved by the National Hospital Organization Fukuoka National Hospital Institutional Review Board for Clinical Research (#F3-9). Informed consent was waived due to the retrospective nature of the study.

## Results

### Demographic and clinical characteristics

Table 1 lists the demographic and clinical characteristics of the study population. The ratio of male to female was about 6:4, and the mean age was 60. The proportions of obese and underweight subjects were 38.5% (n = 20) and 11.5% (n = 6), respectively. Among 8 cases with axial CT images at the navel level, Pearson’s correlation coefficients between the radiological variables used in the current analyses and those at the navel level were all high (> 0.80) (Additional file 1: Table S1).

**Table 1:** Mean Values or Frequencies of Demographic and Clinical Characteristics

Variables	Mean values (standard deviations) or frequencies
Male sex (%)	62.3
Age (years)	60 (20)
Body mass index (kg/m^2^)^a^	23.9 (4.9)
Obesity (%)^a^	38.5
Underweight (%)^a^	11.5
Smokers (%)	50.9
Hypertension (%)	32.1
Diabetes (%)	11.3
*Assessment of abdominal fat component*	
VAT area (cm^2^)	130.7 (89.5)
SAT area (cm^2^)	86.8 (51.7)
TAT area (cm^2^)	217.5 (120.2)
VAT/TAT (%)	56.5 (19.7)
Waist circumference (cm)	88.3 (11.1)

Values are given as means with standard deviations in parentheses for continuous variables and as percentages for dichotomized and categorical variables

*VAT* visceral adipose tissue, *SAT* subcutaneous adipose tissue, *TAT* total adipose tissue

^a^Mean values or proportions were calculated among 52 cases due to the exclusion of 1 case with no available data on body mass index

### Obesity-related variables and COVID-19 progression

A total of 15 and 9 cases (28.3% and 17.0% of the whole study subjects) progressed to severe and critical stages, respectively. The incidences of severe and critical development increased significantly with VAT/TAT (HR per 1% increase = 1.040 (95% CI 1.008–1.074), *P* = 0.01, and HR per 1% increase = 1.070 (95% CI 1.017–1.126), *P* = 0.009, respectively). After adjustment for potential confounders, the positive association of VAT/TAT with COVID-19 aggravation remained significant (HR = 1.055 (95% CI 1.000–1.112) per 1% increase, *P* = 0.049 for being severe; HR = 1.094 (95% CI 1.007–1.187) per 1% increase, *P* = 0.03 for being critical). The other variables were not significantly associated with disease progression (all *P* ≥ 0.24 in multivariate analyses) (Tables 2, 3).

**Table 2:** Hazard Ratios of Obesity-Related Variables for Progression to Severe-Stage Coronavirus Disease 2019

Obesity-related variables	Crude analysis		Multivariable-adjusted analysis^a^	
	HR (95% CI)	*P* value	HR (95% CI)	*P* value
Body mass index (per 1 kg/m^2^ increase)^b^	0.975 (0.874–1.087)	0.64	0.976 (0.845–1.127)	0.74
VAT area (per 1 cm^2^ increase)	1.003 (0.998–1.008)	0.23	1.003 (0.996–1.010)	0.37
SAT area (per 1 cm^2^ increase)	0.994 (0.983–1.006)	0.31	0.999 (0.986–1.012)	0.85
TAT area (per 1 cm^2^ increase)	1.001 (0.997–1.005)	0.65	1.001 (0.997–1.006)	0.57
VAT/TAT (per 1% increase)	1.040 (1.008–1.074)	0.01	1.055 (1.000–1.112)	0.049
Waist circumference (per 1 cm increase)	1.009 (0.965–1.054)	0.70	1.002 (0.941–1.068)	0.94

*HR* hazard ratio, *95% CI* 95% confidence interval, *VAT* visceral adipose tissue, *SAT* subcutaneous adipose tissue, *TAT* total adipose tissue

^a^Adjustment was made for age, sex, smoking status, hypertension, and diabetes

^b^After excluding 1 case with no available data on body mass index, HR was estimated using the remaining 52 cases

**Table 3:** Hazard Ratios of Obesity-Related Variables for Progression to Critical-Stage Coronavirus Disease 2019

Obesity-related variables	Crude analysis			Multivariable-adjusted analysis^a^	
	HR (95% CI)	*P* value		HR (95% CI)	*P* value
Body mass index (per 1 kg/m^2^ increase)^b^	0.965 (0.832–1.118)	0.63		0.902 (0.714–1.140)	0.39
VAT area (per 1 cm^2^ increase)	1.004 (0.997–1.011)	0.25		1.003 (0.995–1.012)	0.45
SAT area (per 1 cm^2^ increase)	0.985 (0.967–1.004)	0.11		0.986 (0.963–1.010)	0.24
TAT area (per 1 cm^2^ increase)	1.000 (0.995–1.006)	0.86		1.000 (0.994–1.007)	0.89
VAT/TAT (per 1% increase)	1.070 (1.017–1.126)	0.009		1.094 (1.007–1.187)	0.03
Waist circumference (per 1 cm increase)	1.013 (0.956–1.073)	0.66		0.993 (0.907–1.086)	0.87

*HR* hazard ratio, *95% CI* 95% confidence interval, *VAT* visceral adipose tissue, *SAT* subcutaneous adipose tissue, *TAT* total adipose tissue

^a^Adjustment was made for age, sex, smoking status, hypertension, and diabetes

^b^After excluding 1 case with no available data on body mass index, HR was estimated using the remaining 52 cases

**Fig. 2:**
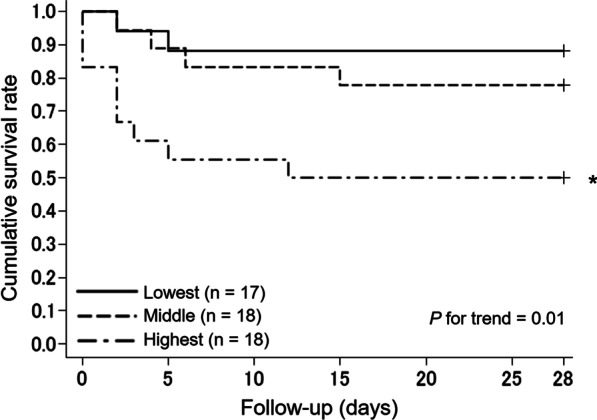
Kaplan–Meier curves for disease progression to severe coronavirus disease 2019 according to the levels of visceral/total adipose tissue. ^*^P < 0.05 versus the reference group. The study subjects were divided into three groups based on the tertile distribution of visceral/total adipose tissue levels as follows: lowest (reference), < 49.0%; middle, 49.0–66.1%; and highest, ≥ 66.2%

**Fig. 3:**
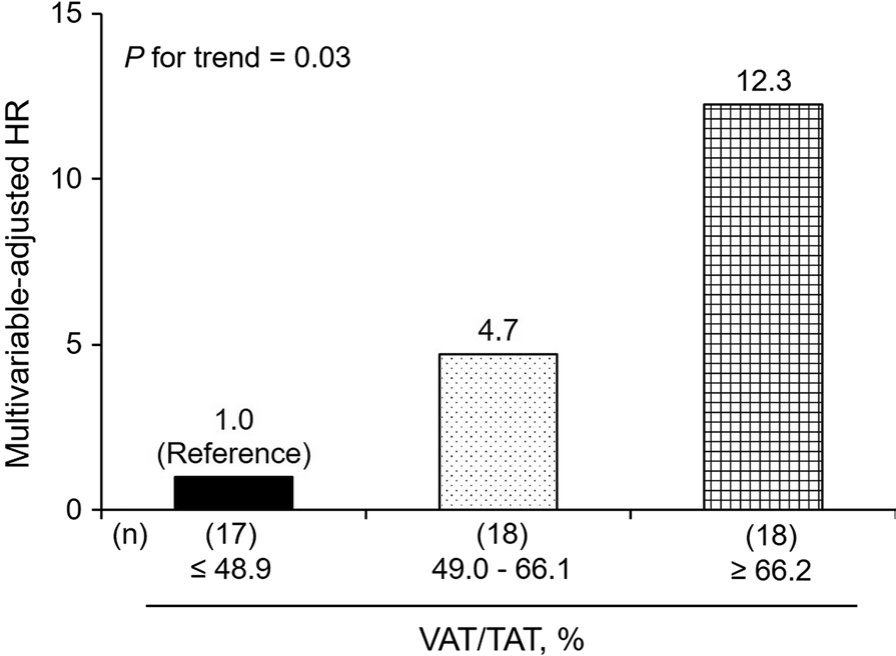
Trends in the multivariable-adjusted HRs for developing severe coronavirus disease 2019 according to the levels of visceral/total adipose tissue. *HR* hazard ratio, *VAT* visceral adipose tissue, *TAT* total adipose tissue. The study subjects were divided into three groups based on the tertile distribution of visceral/total adipose tissue levels as follows: lowest (reference), < 49.0%; middle, 49.0–66.1%; and highest, ≥ 66.2%. Adjustment was made for age, sex, smoking status, hypertension, and diabetes.

When classifying the subjects into three groups, the incidence of developing severe COVID-19 increased in cases with higher VAT/TAT levels (*P* = 0.01 for trend). In comparison with the lowest tertile group, the HR was significantly higher in the highest tertile group (HR = 5.78 (1.25–26.82), *P* = 0.03) (Fig. 2). The linear trend remained significant after multivariable adjustment (*P* = 0.03), with marginal significance in the highest versus lowest tertile group (HR = 11.96 (0.96–149.21), *P* = 0.05) (Fig. 3). Limiting the study endpoint to progression toward critical illness did not alter the observed outcomes (*P* = 0.02 for trend) (Additional file 1: Fig. S1). The results were similar when analyzed separately for smokers and never smokers (Additional file 1: Table S2, Fig. S2, and Fig. S3). As to BMI-based categories (underweight / normal / obese), the trend in the risk of an unfavorable course did not reach statistical significance, with a somewhat higher risk in the underweight and obese categories than in the normal BMI category (Additional file 1: Fig. S4).

### Prediction ability of VAT/TAT and BMI

**Fig. 4:**
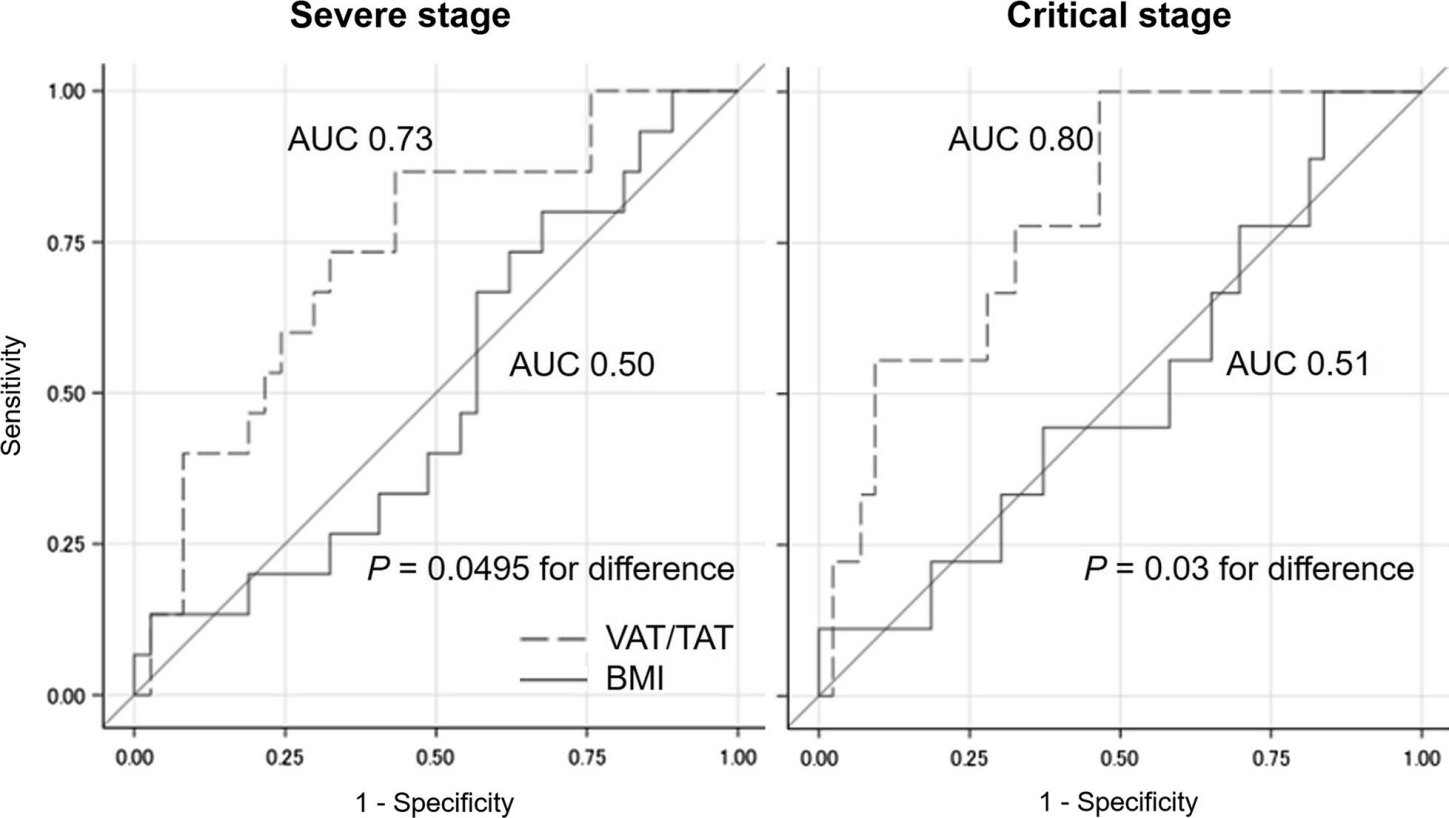
Receiver operating characteristic curves for developing severe or critical coronavirus disease 2019. *VAT* visceral adipose tissue, *TAT* total adipose tissue, *BMI* body mass index, *AUC* area under the receiver operating characteristic curve. Regarding BMI, analyses were performed using 52 cases due to the exclusion of 1 case with no available data on body mass index.

Regarding COVID-19 progression to the severe stage, the predicting ability of VAT/TAT was significantly better than that of BMI (AUC of 0.73 for VAT/TAT and 0.50 for BMI; *P* = 0.0495 for the difference). Broadly similar results were obtained after replacement of the prediction objective to developing critical COVID-19 (AUC of 0.80 for VAT/TAT and 0.51 for BMI; *P* = 0.03 for the difference) (Fig. 4).

## Discussion

The present cohort study revealed that VAT/TAT elevation was a significant risk factor for progression to severe or critical disease in COVID-19 patients. The significant trends were also observed in the association of the VAT/TAT levels with the future risk of COVID-19 progression. ROC analysis showed the significant superiority of VAT/TAT to BMI for predicting morbidity in COVID-19. The outcomes did not vary by smoking status. This is the first time-to-event analysis to evaluate the prognostic impact of VAT/TAT on COVID-19.

To the best of our knowledge, there have been only four studies investigating obesity-related radiological biomarkers for prognosis in subjects with COVID-19 [12–15], all of which were conducted in Italy or the United States. The present study is the first to evaluate this issue in the Asia–Pacific region. The previous ones showed the association of VAT with the likelihood of intensive care unit admission or developing pneumonia; however, all of them were designed as retrospective or cross-sectional studies, and the authors were unable to make a causal inference. The present research was unique in controlling for potential confounders to highlight the robustness of the results. Our findings reinforced and ensured the value of intra-abdominal fat mass as an independent risk factor for COVID-19 progression.

VAT/TAT can increase directly on account of VAT overload and/or relatively due to frailty [16]. Independently of coexistent metabolic disorders such as diabetes and cardiovascular disease, excessive adipose tissue increases the risk of SARS-CoV-2 infection via enhancement of viral entry due to hyperexpression of angiotensin-converting enzyme 2 [17, 18], cell surface glucose–regulated protein 78 [19, 20], heparan sulfate proteoglycan [21, 22], and neuropilin-1 [23, 24]. Adipose tissue and its related immune cells also induce an inadequate response and overreaction of the immune system, triggering a cytokine storm [25, 26]. Furthermore, in the obese, the risk for developing a thrombus formation and hemorrhage is elevated due to hyperleptinemia [27], the upregulation of plasminogen activator inhibitor 1 [28], endothelial dysfunction [29], and the impaired bioavailability of vitamin K [30]. On the other hand, frailty has been widely known as a coexisting condition with underweight and high intra-abdominal fat mass [16], causing high vulnerability for dependency and death [31]. A recent multicenter observational cohort study revealed that frailty raised the risk of morbidity and mortality in patients with COVID-19 [32]. Hence, it is reasonable for VAT/TAT, rather than BMI, to be strongly correlated with unfavorable outcomes in COVID-19, as demonstrated in the present study.

Several studies have indicated that a high BMI, namely, overweight and obesity, is a risk factor for major complications in COVID-19 [5, 6, 33]. In line with the previous report [14], our analysis showed the reliable performance of VAT/TAT, distinct from BMI, as a prognostic factor in COVID-19. The differences in predictive adequacy between VAT/TAT and BMI were likely to be affected by the aforementioned influence of frailty on the prognosis of COVID-19, since frailty is linked to high VAT/TAT and low BMI [16]. The association of frailty with poor prognosis was parallel to that of VAT/TAT, whereas it was inconsistent with the undesirable effect of increased BMI and therefore seemed to have attenuated the trend in the relationship between BMI and disease progression in COVID-19. The current results confirmed the usefulness and importance of VAT/TAT assessment in the field of COVID-19.

The strengths of our study were the highly accurate measurement of intra-abdominal fat deposition based on CT images, the time-to-event design to minimize the effects of reverse causation, and the certainty of the findings established through multivariable adjustment and stratified analyses. However, some potential limitations should be noted. First, the value of VAT/TAT was based on a single measurement. Second, VAT/TAT and other radiological biomarkers were estimated using a CT image at the level of upper pole of the right kidney, not at the navel or L4/L5 level, since there were no data from axial CT images at those levels for most of the study subjects. These two limitations might have caused misclassification of the VAT/TAT level, which could have weakened the association found in the present study, biasing the results toward a null hypothesis. However, strong correlations were demonstrated between radiological factors at the upper pole of the right kidney and those at the navel level in the present study, consistent with preceding literature [34, 35]. Thus, the value of VAT/TAT at the upper pole of the right kidney, usually available from routinely collected chest CT, appeared comparable to the value at the standard position and is practical for use in daily clinics. Third, we were unable to adjust the effects of intensity or duration of smoking on the prognosis of COVID-19 due to a lack of data concerning the number of pack years of cigarette smoking. However, in a subgroup analysis by smoking status, the association of VAT/TAT with the risk of COVID-19 progression did not differ from the primary results. We therefore speculate that this limitation did not alter our conclusions. Fourth, BMI was calculated using the values of self-reported height and weight. It might have been underestimated to some extent, since the BMI value based on self-reported height and weight was reported to be 0.2–1.8 kg/m^2^ lower than that based on measured height and weight [36]. Fifth, hypertension and diabetes might have been overlooked, since they were not diagnosed by the measurement of blood pressure or glucose levels on admission, both of which could be elevated due to systemic inflammation from COVID-19. Lastly, the sample size was relatively small owing to the study design as single-center analyses. This limitation might have led to overlooking the association between obesity-related biomarkers other than VAT/TAT and the endpoint of interest. To overcome this limitation, a larger-scale cohort study is warranted.

## Conclusions

An elevated VAT/TAT is an independent risk factor for disease progression among COVID-19 patients. VAT/TAT has advantages over BMI in prediction ability for COVID-19 morbidity. Our findings suggest that chest CT of COVID-19 cases should be assessed not only for the presence of pneumonia but also for the level of VAT/TAT and that COVID-19 patients with increased VAT/TAT levels should be carefully observed as high-risk individuals for morbidity and mortality.

## Supplementary Information

**Additional file 1.** Additional figures and tables.

## Data Availability

The datasets generated and/or analysed during the current study are not publicly available due to limitations of ethical approval involving the patient data and anonymity but are available from the corresponding author on reasonable request.

## Abbreviations

SARS-CoV-2: Severe acute respiratory syndrome coronavirus 2
COVID-19: Coronavirus disease 2019
BMI: Body mass index
CT: Computed tomography
VAT: Visceral adipose tissue
SAT: Subcutaneous adipose tissue
TAT: Total adipose tissue
SpO_2_: Oxygen saturation
HR: Hazard ratio
95% CIs: 95% Confidence intervals
ROC: Receiver operating characteristic
AUC: Area under the ROC curve
