# Supplementary material for: Impact of visceral fat on the prognosis of coronavirus disease 2019: an observational cohort study
Source: BMC Infect Dis. 2021 Dec 10;21:1240. doi: 10.1186/s12879-021-06958-z (PMC8660963; doi:10.1186/s12879-021-06958-z)
Supplement: Supplementary file 1 — Additional file 1. Additional figures and tables. [file 12879_2021_6958_MOESM1_ESM.docx]

**Additional file 1**

**Table S1.** Pearson’s Correlation Coefficients for Radiological Variables between Those at the Upper Pole of the Right Kidney and Those at the Navel Level

| Variables | Pearson’s correlation coefficient | *P* value |
| --- | --- | --- |
| VAT area | 0.89 | < 0.01 |
| SAT area | 0.81 | 0.02 |
| TAT area | 0.87 | < 0.01 |
| Waist circumference | 0.97 | < 0.01 |

**Table S2.** Hazard Ratios per 1% Increase of VAT/TAT for Progression to Severe- or Critical-Stage Coronavirus Disease 2019

| Study endpoint | Crude analysis | |  | Multivariable-adjusted analysis^*^ | |
| --- | --- | --- | --- | --- | --- |
|  | HR (95% CI) | *P* value |  | HR (95% CI) | *P* value |
| Smokers (n = 27) |  |  |  |  |  |
| Severe stage | 1.047 (0.992-1.104) | 0.09 |  | 1.024 (0.957-1.095) | 0.50 |
| Critical stage | 1.064 (0.982-1.153) | 0.13 |  | 1.022 (0.932-1.120) | 0.64 |
|  |  |  |  |  |  |
| Never smokers (n = 26) |  |  |  |  |  |
| Severe stage | 1.042 (0.994-1.092) | 0.09 |  | 1.184 (1.025-1.369) | 0.02 |
| Critical stage | 1.101 (1.012-1.197) | 0.02 |  | 1.267 (0.966-1.661) | 0.09 |

HR, hazard ratio; 95% CI, 95% confidence interval; VAT, visceral adipose tissue; TAT, total adipose tissue.

^*^Adjustment was made for age, sex, hypertension, and diabetes.

**Figure S1.** Kaplan–Meier curves for disease progression to critical coronavirus disease 2019 according to the levels of visceral/total adipose tissue.


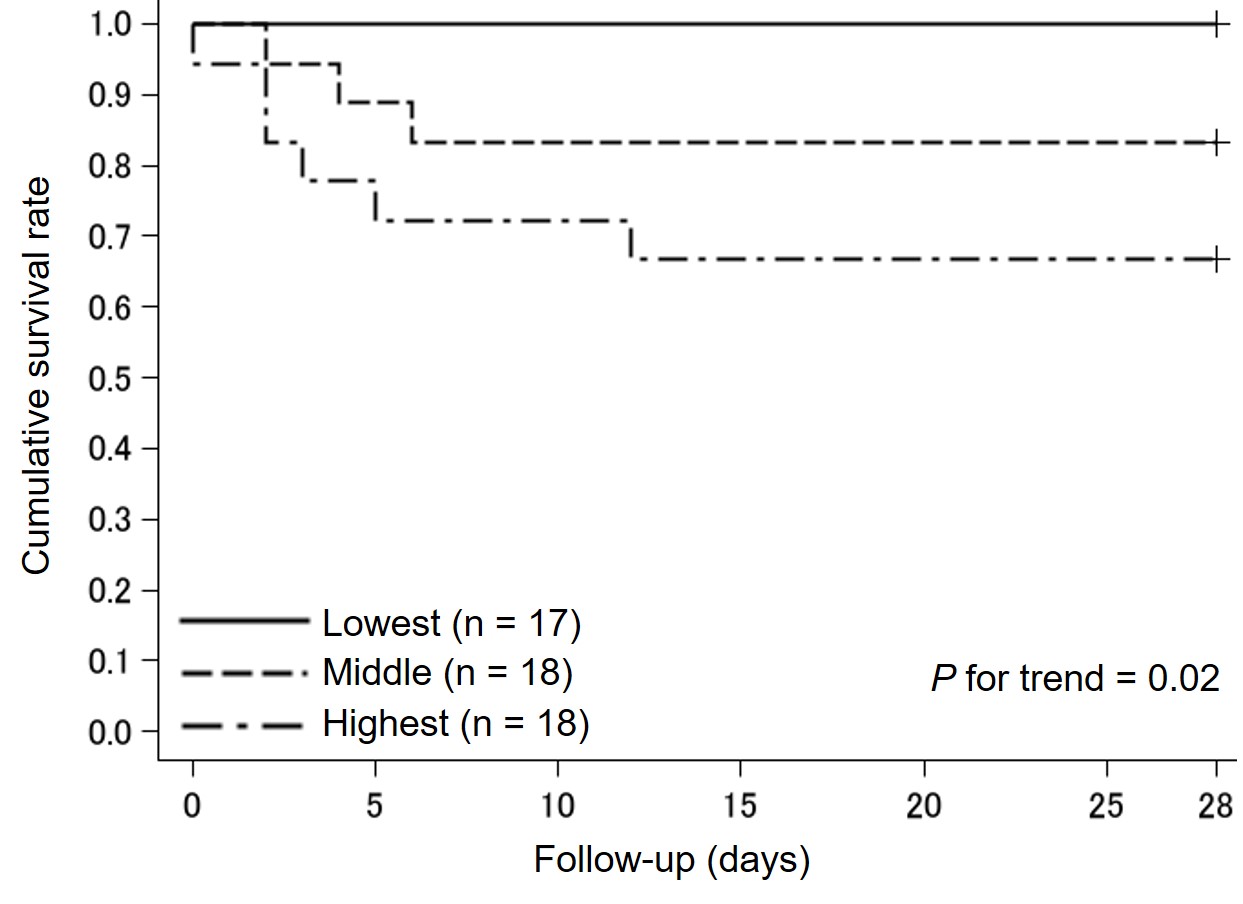


The study subjects were divided into three groups based on the tertile distribution of visceral/total adipose tissue levels as follows: lowest, < 49.0%; middle, 49.0–66.1%; and highest, ≥ 66.2%.

**Figure S2.** Kaplan–Meier curves for disease progression to severe or critical coronavirus disease 2019 according to the levels of visceral/total adipose tissue among never smokers.


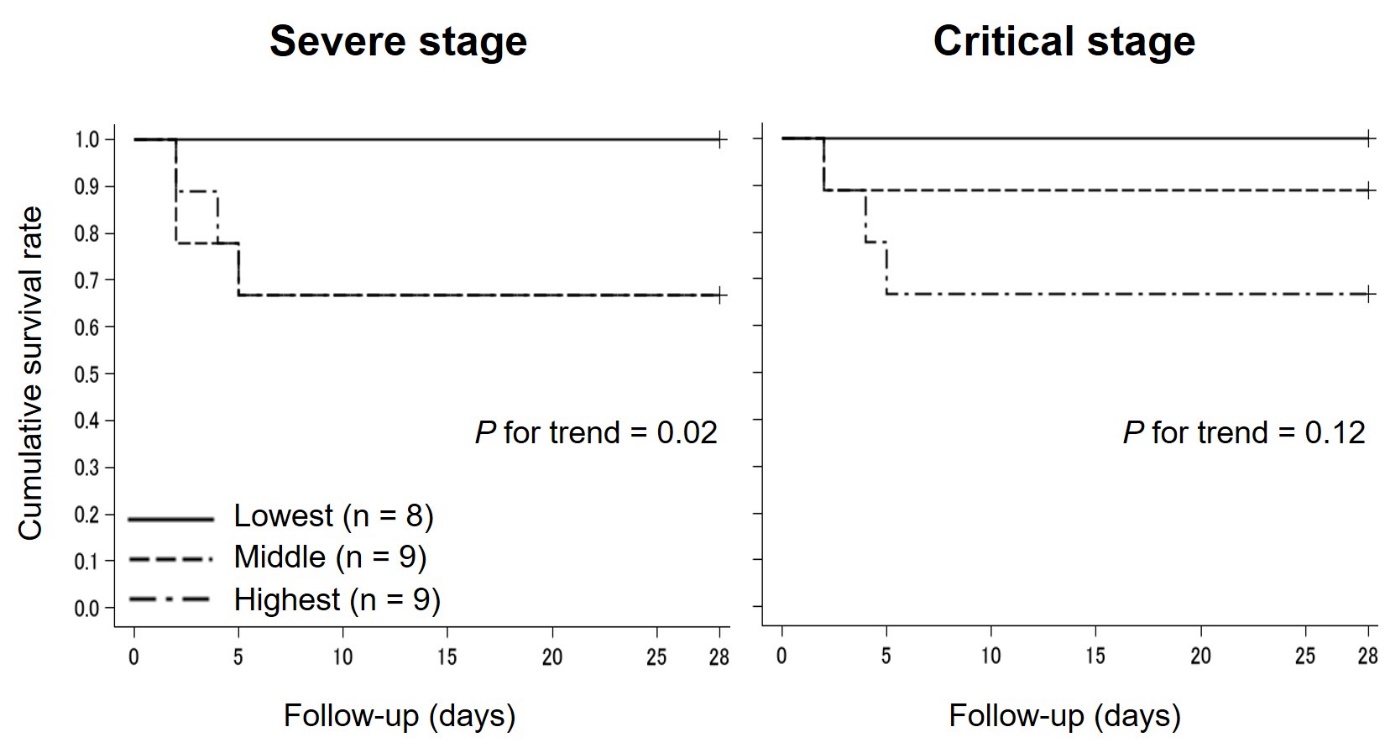


The study subjects were divided into three groups based on the tertile distribution of visceral/total adipose tissue levels as follows: lowest, < 33.0%; middle, 33.0–57.9%; and highest, ≥ 58.0%.

**Figure S3.** Kaplan–Meier curves for disease progression to severe or critical coronavirus disease 2019 according to the levels of visceral/total adipose tissue among smokers.


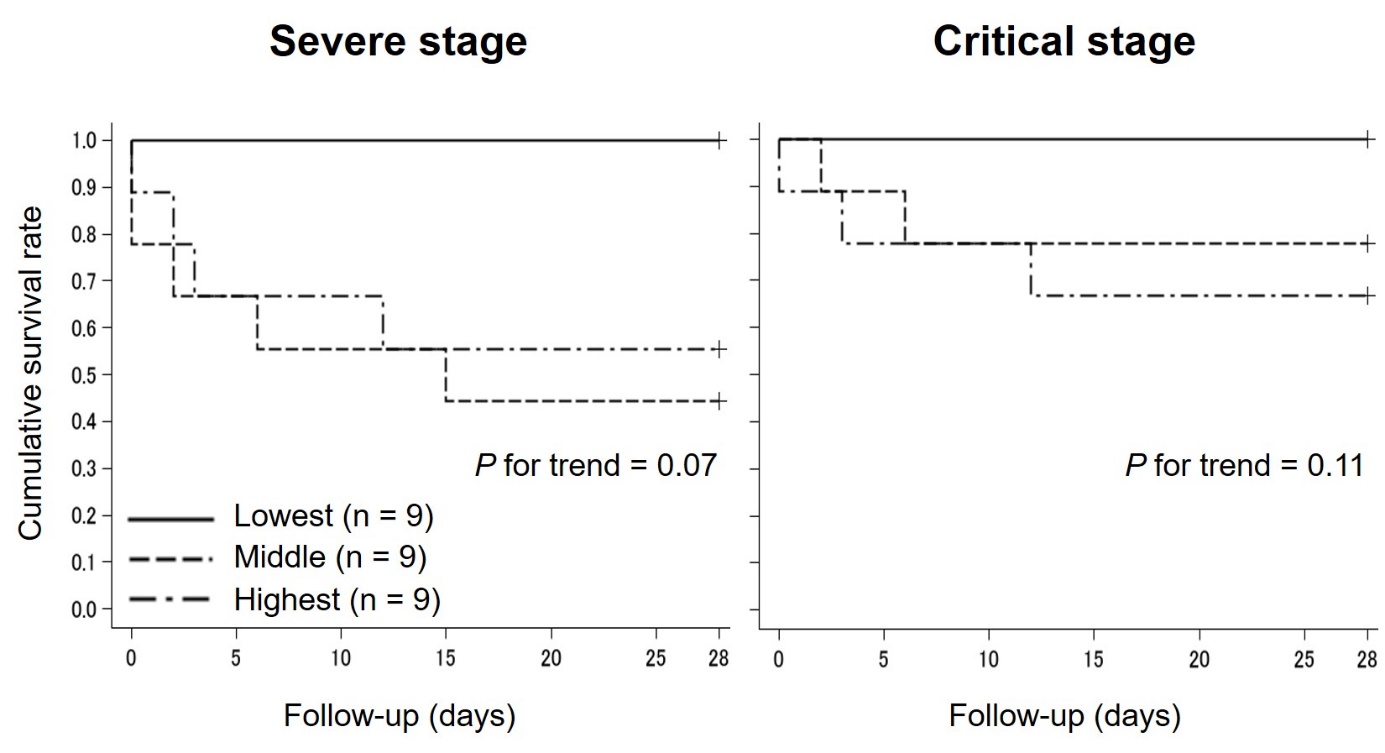


The study subjects were divided into three groups based on the tertile distribution of visceral/total adipose tissue levels as follows: lowest, < 60.0%; middle, 60.0–74.9%; and highest, ≥ 75.0%.

**Figure S4.** Kaplan–Meier curves for disease progression to severe or critical coronavirus disease 2019 according to the levels of body mass index.


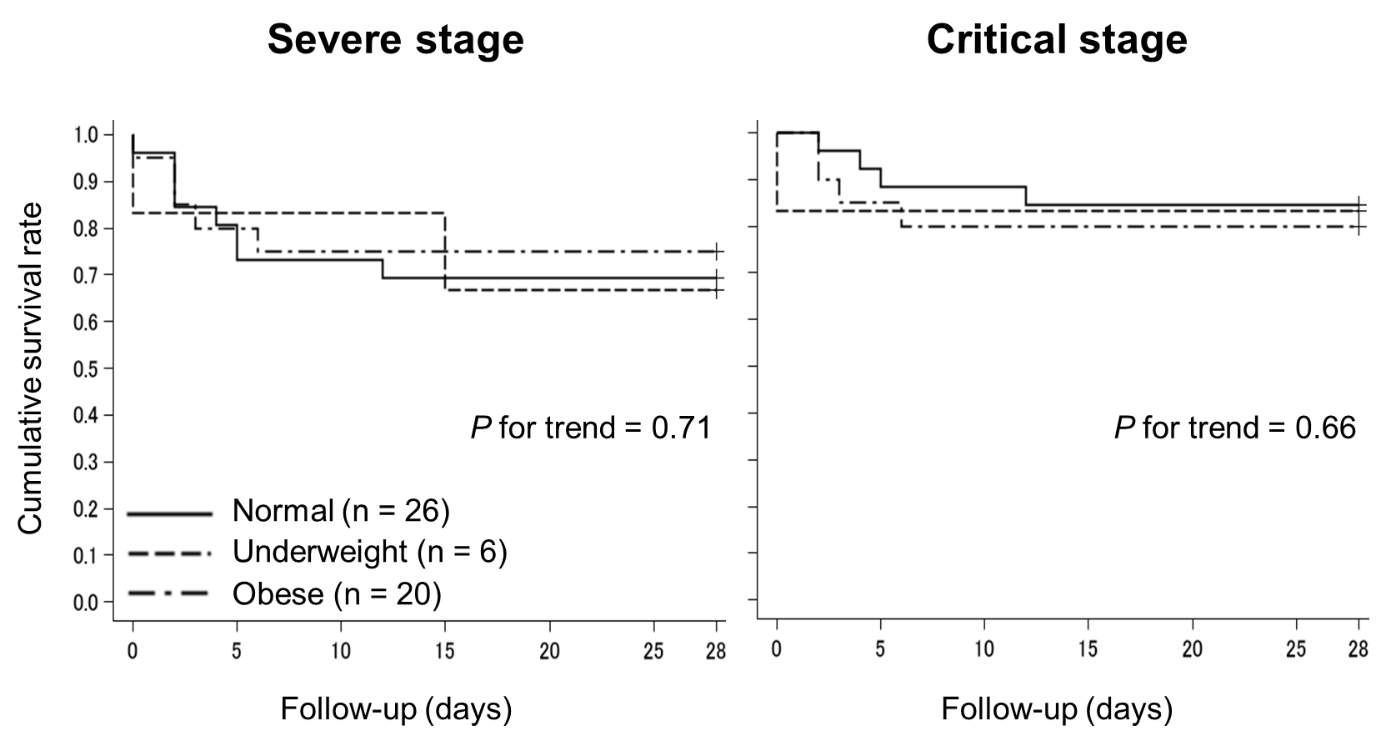


BMI, body mass index.

The analyses were performed using 52 cases due to the exclusion of 1 case with no available data on BMI.

The study subjects were divided into three groups based on the values of body mass index as follows: normal, ≥ 18.5 and < 25.0 kg/m^2^; underweight, < 18.5 kg/m^2^; and obese, ≥ 25.0 kg/m^2^.
